# Supplementary material for: Ultrasensitive and high-efficiency screen of de novo low-frequency mutations by o2n-seq
Source: Nat Commun. 2017 May 22;8:15335. doi: 10.1038/ncomms15335 (PMC5458117; doi:10.1038/ncomms15335)
Supplement: Supplementary Information — Supplementary Figures [file ncomms15335-s1.pdf]

```
1, A-tailing DNA
                    5'   --------DNA---------A   3'
                    3'  A--------DNA---------    5'
2, Y-adaptor
                   5'  pGATCAGTCGTACGTGCTTACTCTCAATAGCAGCTT  3'
                   3'  TCUAGTCAGCA
                                  AGTGGCTGACGGGTGp  5'
3, Ligation
              5'  pGTGGGCAGTCGGTGA
                                  ACGACTGAUCT--------DNA---------AGATCAGTCGTACGTGCTTACTCTCAATAGCAGCTT  3'
     3'  TTCGACGATAACTCTCATTGCTGCATGCTGACTAGA--------DNA---------TCUAGTCAGCA
                                                                          AGTGGCTGACGGGTGp  5'

3, Circularization

                 |----------------------------<---------------------------------------------------|
                 |-GTGGGCAGTCGGTGA                                                                |
                                 ACGACTGAUCT--->----DNA---------AGATCAGTCGTACGTGCTTACTCTCAATAGCAGCTT-


5, Second strand synthesis and nicking
             |---------------->----------------------------------------------->----------------------|
             | |-----------------------------------------<----------------------------------------| |
             | |                                                                                  | |
             | --GTGGGCAGTCGGTGAACGACTGA CT->------DNA----------AGATCAGTCGTACGTGCTTACTCTCAATAGCAGCTT-  |
             |---CACCCGTCAGCCACTTGCTGACTAGA--------DNA--------<-TCTAG  5'         ATGAGAGTTATCGTCGAA--|


6, Strand displacement

5'   GATCT----------DNA----------AGATCAGTCGTTCACCGACTGCCCACAAGCTGCTATTGAGAGTAAGCACGTACGACTGATCT----------DNA----------AG  3'
3'   CTAGA----------DNA----------TCTAGTCAGCAAGTGGCTGACGGGTGTTCGACGATAACTCTCATTCGTGCATGCTGACTAGA----------DNA----------TC  5'
     |5bp|----------DNA----------|-------------------62bp-----------------------------------|----------DNA---------|2bp|
```

Supplementary Figure 1. Schematic diagram of o2n-seq. Double-stranded DNA was sheared, end-repaired, and underwent A-tailing by standard protocols. A-tailed DNA was ligated to T-tailed dUTP adapters, circularized using single-strand DNA ligase, and used for second strand synthesis followed by strand displacement amplification. The amplified DNA is shown as step 6, and was subsequently used to prepare the standard NGS libraries.

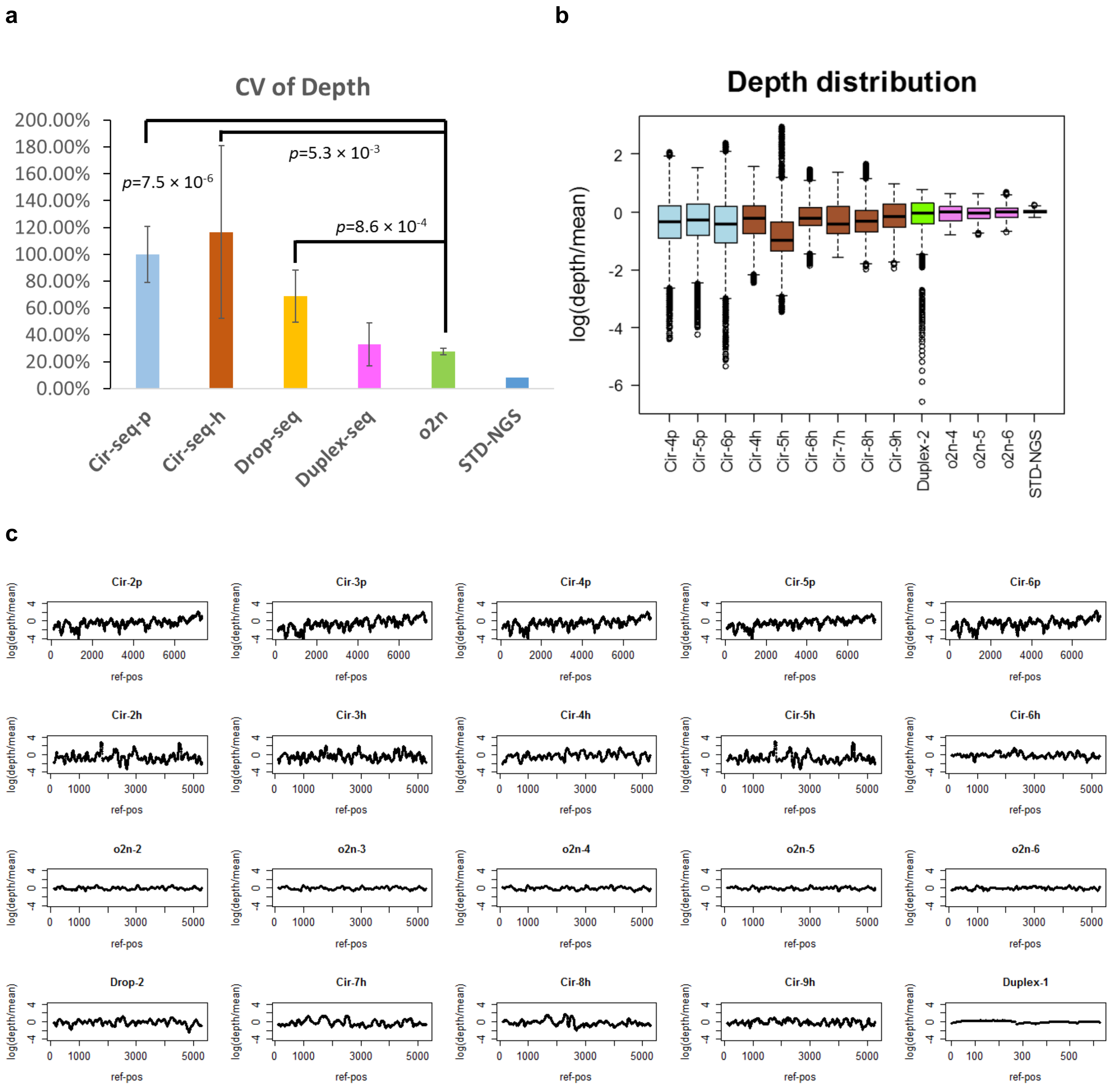

Supplementary Figure 2. Read depth distribution of Cir-seq, Duplex-seq, o2n-seq, and Droplet-CirSeq libraries. **(a)** Read depth CV (coefficient of variance) for Cir-seq for poliovirus, Cir-seq for *phix174*, Droplet-CirSeq, o2n-seq, and STD-NGS libraries (means ±s.d.). The o2n-seq libraries exhibited a depth CV 3.6 times lower than that of Cir-seq for poliovirus libraries, 4.22 times lower than that of Cir-seq for *phix174* libraries and 2.5 times lower than that of Droplet-CirSeq libraries. The o2n-seq library depth CV was closer to that of the standard NGS library. Two-tailed Student's t-test was used for statistical analysis. **(b)** Boxplot of read depth distribution. Y-axis represents the log(e) ratio of depth over mean of the genome. Three replicates of Cir-seq-poliovirus, three replicates of Cir-seq-*phix174*, one replicate of Duplex-seq, three replicates of o2n-seq, and one standard NGS (insert size: 90 bp) were plotted. **(c)** Read depth distribution of Cir-seq-poliovirus, Cir-seq-*phix*174, Droplet-CirSeq and o2n-seq. This figure showed the o2n-seq had a more highly concentrated read depth distribution than Cir-seq and Droplet-seq, and each site depth for o2n-seq was closer to the mean value of depth.

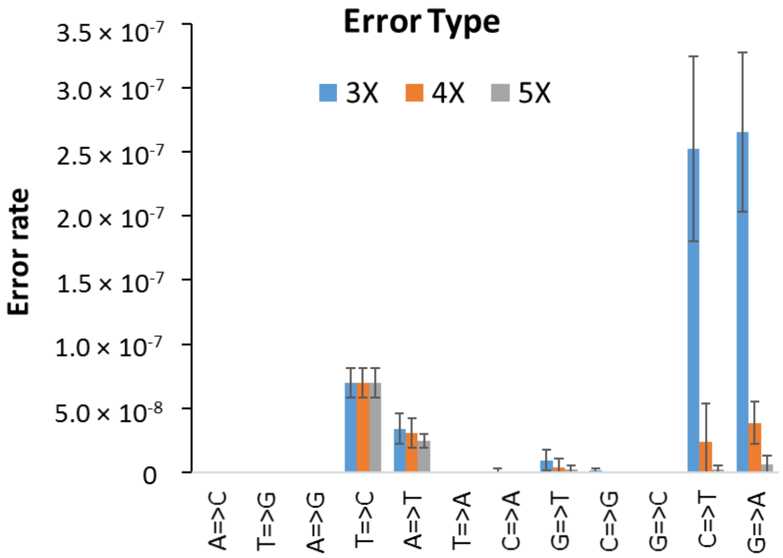

Supplementary Figure 3. Mutation types obtained by o2n-seq for 3X, 4X or 5X CSs (three experimental replicates, means ±s.d.).

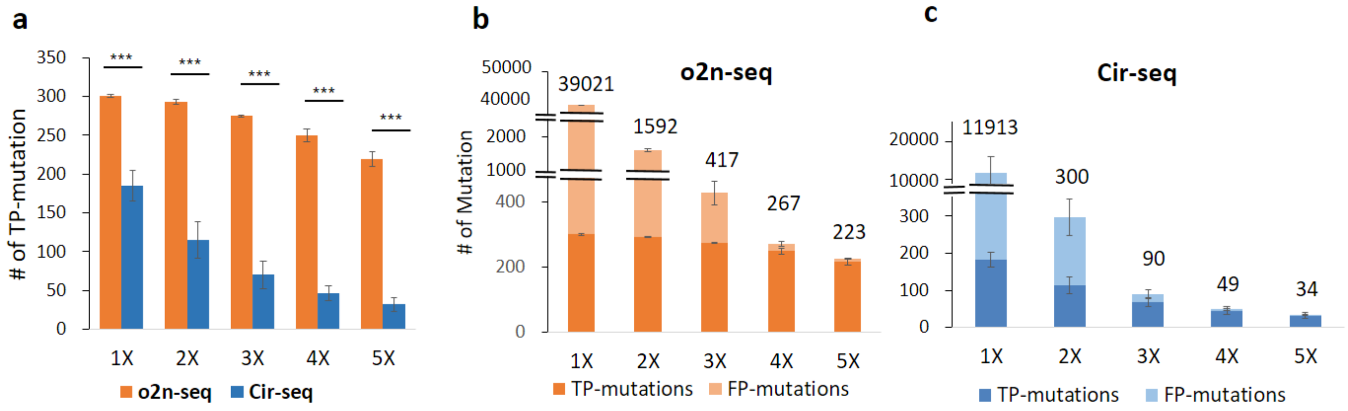

Supplementary Figure 4. Number of mutations detected by o2n-seq and Cir-seq. **(a)** Number of true positive (TP) mutations detected using o2n-seq (three experimental replicates, means ±s.d.) and Cir-seq (three experimental replicates, means ±s.d.) under different CSs criteria. Two-tailed Student's t-test was used for statistical analysis. **(b, c)** Total mutations, including both TP and FP mutations, detected by o2n-seq (three experimental replicates, means ±s.d.) and Cir-seq (three experimental replicates, means ±s.d.). Two-tailed Student's t-test was used for statistical analysis.

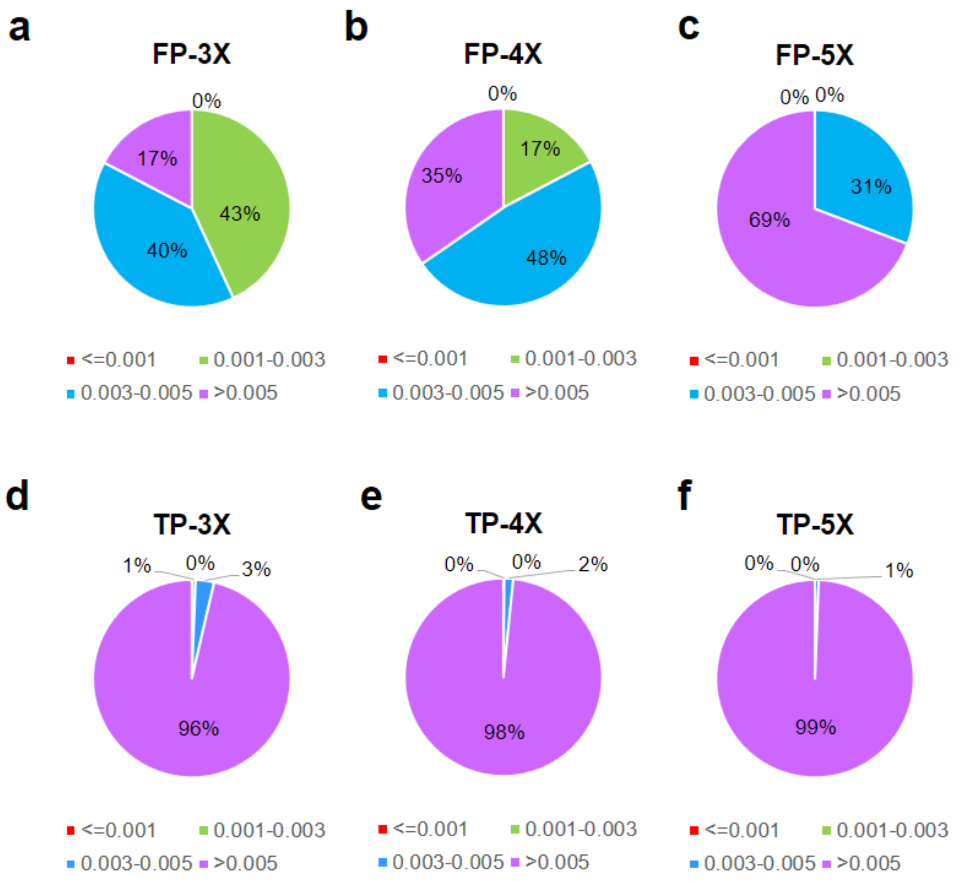

Supplementary Figure 5. Mutation frequency distribution of FP and TP variants detected by o2n-seq under different CSs (3X-5X) for the 1:100 mixture of *E.coli.*

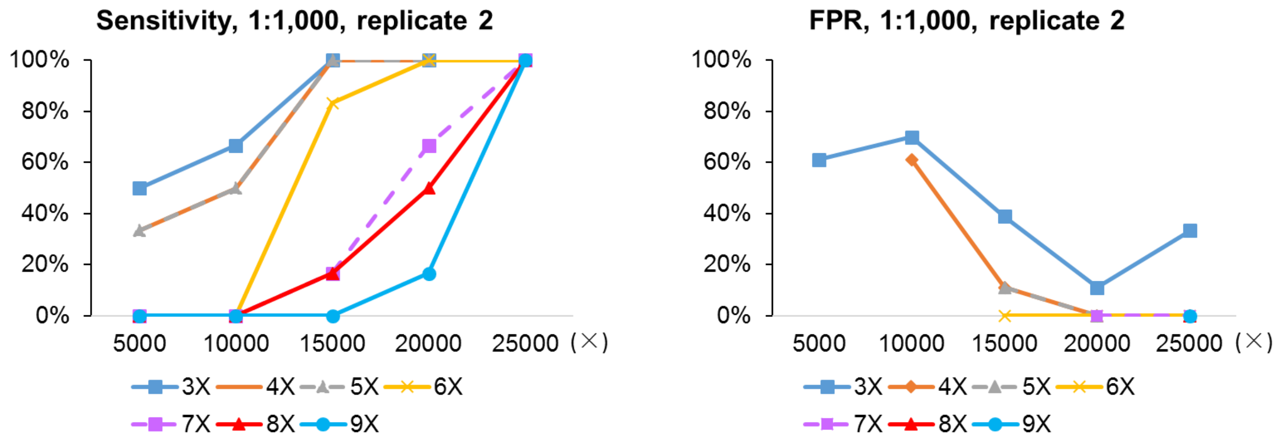

Supplementary Figure 6. Sensitivity and FPR of mutation detection of o2n-seq by different CSs criteria (3X-9X) under different total CSs coverage (5,000-25,000 ×) for the 1: 1,000 mix of *phix174* (the other experimental replicate). Dash lines were used to display the overlapped results.

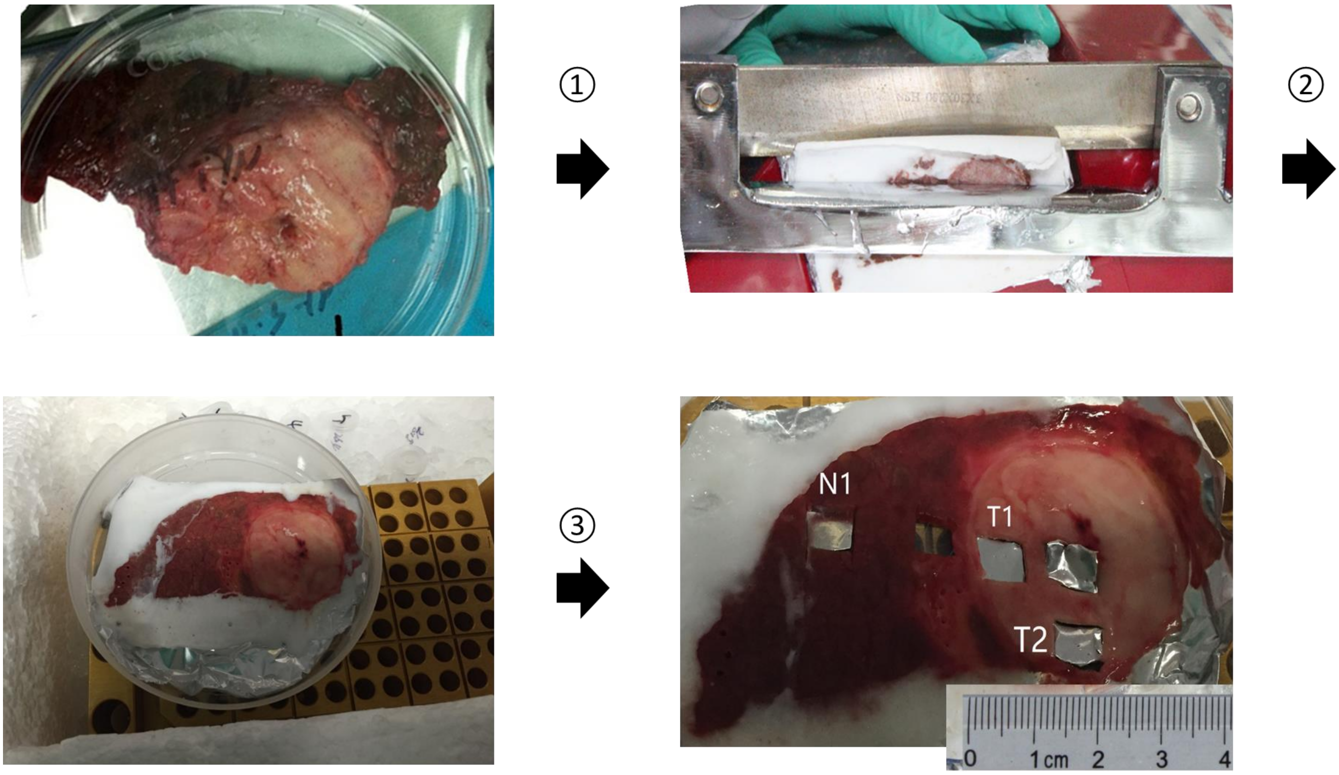

Supplementary Figure 7. Samples collection. Tumor tissues were embedded in O.C.T. and sliced into 1-mm-thick pieces. One normal sample (N1) two tumor samples (T1, T2) were collected from one 1-mm-thick slice.

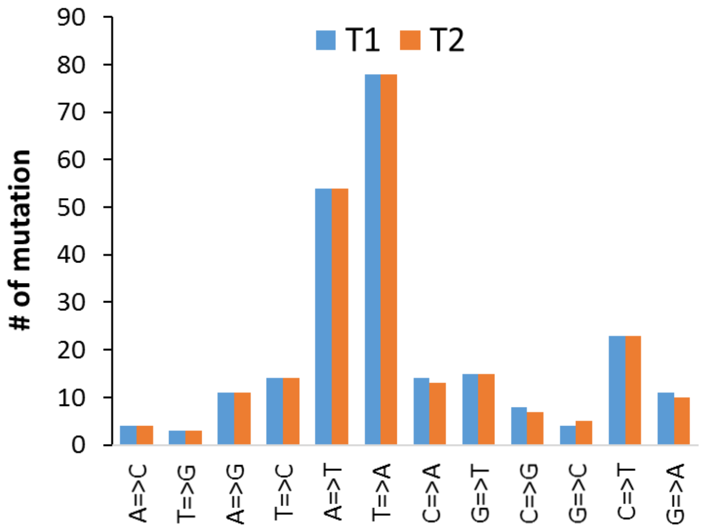

Supplementary Figure 8. Distribution of mutation type for high frequency somatic mutations in tumor samples (T1 and T2).

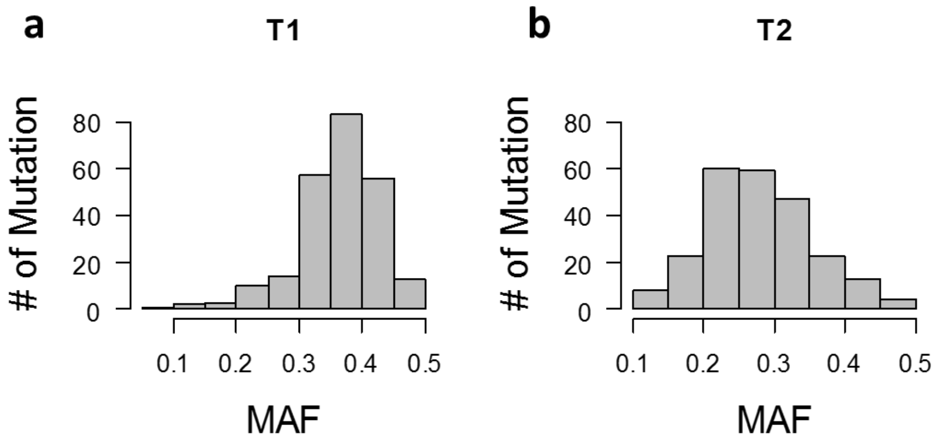

Supplementary Figure 9. The MAF (detected by VarScan) of high frequency somatic mutation distribution for tumor samples (T1 and T2). Different MAF patterns for different samples suggest different heterogeneity levels.

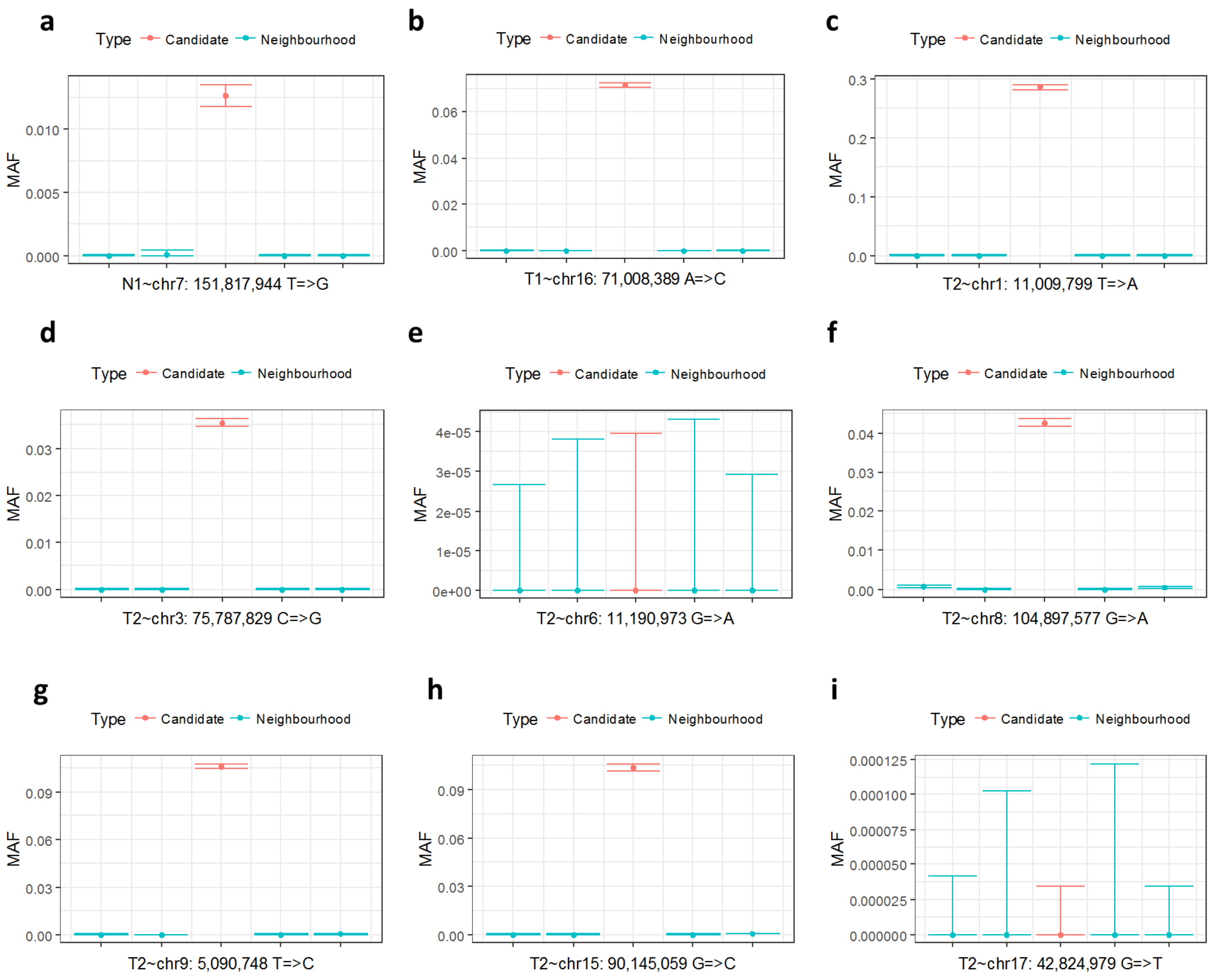

Supplementary Figure 10. Validation of low-frequency mutations by ultra-deep amplicon sequencing for N1, T1, T2, and T3. The x-axis represents ± 2 bp homozygous neighbors (blue) and the candidate locus (red). The y-axis represents the MAF of mutations.

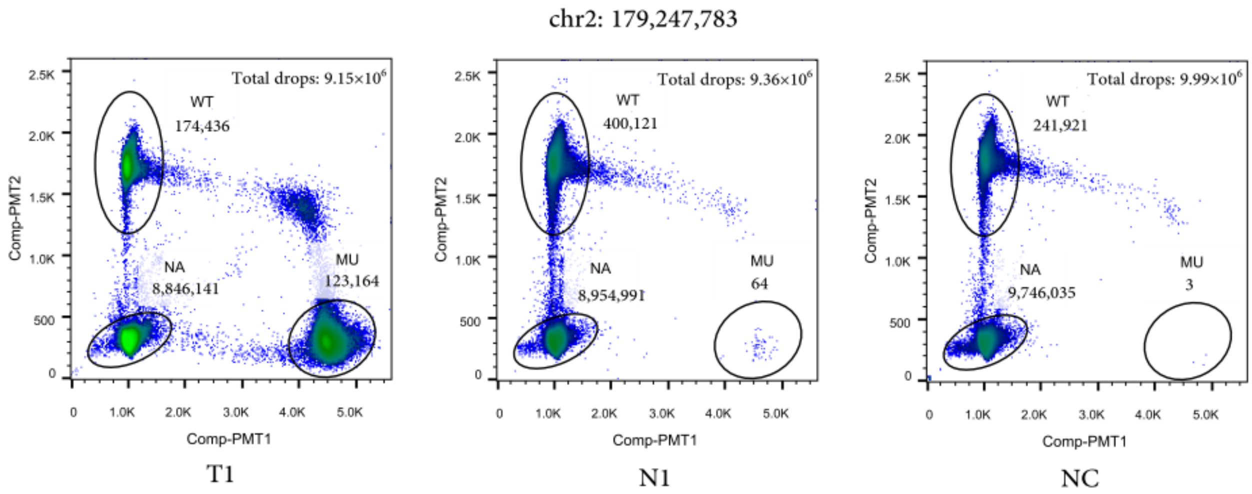

Supplementary Figure 11. Validated ultralow-frequency mutations in normal sample by digital droplet PCR. T1 was chosen as the positive control (frequency of mutant allele: 40%). N1 is the sample and NC is the negative control (normal individual). Signal from a single droplet is one dot on the graph. Comp-PTM1 represents the signal for the mutant allele (MU), and Comp-PTM2 represents the signal for the wild-type (WT) allele. NA represents the droplets that do not contain any probe targets. Numbers below the WT/MU/NA labels are the droplet number with corresponding signal.
